# The Aurora-Kinase A Phe31-Ile polymorphism as possible predictor of response to treatment in head and neck squamous cell carcinoma

**DOI:** 10.18632/oncotarget.24355

**Published:** 2018-01-30

**Authors:** Alexander Baumann, Anna Maria S. Buchberger, Guido Piontek, Dominik Schüttler, Martina Rudelius, Rudolf Reiter, Lena Gebel, Gerhard Piendl, Gero Brockhoff, Anja Pickhard

**Affiliations:** ^1^ Department of Otolaryngology Head and Neck Surgery, Helios Amper-Klinikum Dachau, Dachau, Germany; ^2^ Department of Otolaryngology Head and Neck Surgery, Technical University of Munich, Munich, Germany; ^3^ Medizinische Klinik und Poliklinik I, University of Munich, Munich, Germany; ^4^ Institute of Pathology, University of Düsseldorf, Düsseldorf, Germany; ^5^ Department of Otolaryngology Head and Neck Surgery, Section of Phoniatrics and Pedaudiology, University of Ulm, Ulm, Germany; ^6^ University Medical Center Regensburg, Department of Gynecology and Obstetrics, Regensburg, Germany

**Keywords:** HNSCC, Aurora-Kinase, Aurora-Kinase a polymorphism, radiation, inhibition

## Abstract

Recently the Aurora-Kinases (Aurk) moved into the focus as novel disease related biomarkers and therapeutic targets. Elevated Aurora-Kinase expression has been found in a number of malignancies, amongst them HNSCC. For esophageal cancer, the AurkA Phe31-Ile polymorphism has previously been associated with tumor progression. Here we evaluated the treatment efficiency of HNSCC cell radiation as a function of Aurora-Kinases in HNSCC cell lines. Moreover, we investigated a potential sensitization to radiation by a cell treatment with the inhibitors Alisertib, Barasertib, Docetaxel and VX-680.

In parallel the radiation dependent expression and regulation of AurkA/B, p-Akt Ser 473 and Survivin and the AurkA polymorphism were investigated in primary tumor samples. We identified a high-risk collective with elevated AurkA and Survivin or AurkA and p-Akt Ser 473 expression.

High AurkA, AurkB, and p-Akt Ser 473 expression was exclusively found in the heterozygous cell line. We found a polymorphism dependent sensitivity to treatments with different Aurk inhibitors: The homozygous cell line UD-SCC-5 could be sensitized to radiation with Docetaxel in combination with any of the Aurora-Kinase inhibitors. In contrast, treatment with Docetaxel or radiation did not enhance the inhibitory effect of Barasertib or VX-680 in the heterozygous SAS cell line.

These findings indicate that the Aurora-Kinase A Phe31-Ile-polymorphism is a possibly predictive factor for response to radiation in combination with Docetaxel and Aurora-Kinase inhibitor treatments.

## INTRODUCTION

The median 5-year survival for patients with head and neck squamous cell carcinoma (HNSCC) is 50% [1] and at least 50% of patients with a stage III disease relapse in the first two years after therapy [2]. These facts reflect the very poor prognosis for patients with HNSCC.

First attempts of HNSCC therapy in the early twentieth century pursued an anatomical approach based on surgical resection and radiotherapy. In recent years, disease specific biomarkers and signal transduction pathways as potential therapy targets have been identified. For example, targeting the epidermal growth factor receptor (EGFR) with cetuximab (Erbitux^®^) has been established and complements standard approaches. It has been shown that EGFR targeting significantly improves the overall survival (OS) of HNSCC patients [1].

Recently the Aurora-Kinases (Aurk) moved into the focus as novel disease related biomarkers and (potential) therapeutic targets. Aurora-Kinases regulate cell division and mitosis by assembling a functional mitotic spindle, which is essential for chromosome segregation and subsequent cytokinesis. The Aurora-Kinase C is mainly expressed in the testicles and supports the Aurora-Kinase B function [3]. Elevated Aurora-Kinase expression has been found in a number of malignancies [4], amongst them HNSCC [5, 6]. Moreover, it has been shown that the AurkA Phe_31_-Ile polymorphism enhances esophageal cancer progression [7]. In HNSCC, this polymorphism seems to affect the response to cetuximab treatment [6].

Aurora-Kinases A and B are considered to predominantly drive tumor progression and to affect the susceptibility to target specific treatments. Here we evaluated the Aurora-Kinase A polymorphism as a predictor for treatment outcome of HNSCC cell lines. To this end, the cells were treated with Docetaxel and with or without Aurk inhibitors in combination with irradiation.

## RESULTS

### AurkA Phe31-Ile polymorphism correlates with the stage of HNSCC patients

The distribution of the AurkA polymorphism in a sub-cohort of 115 HNSCC patients was evaluated. There was no statistically significant correlation of the AurkA polymorphism and patient's survival (disease free survival *p* = 0.671, overall survival *p* = 0.783). Also, there was no correlation with the T-, N-, or M-classification.

### AurkA expression identifies high risk groups

**Figure 1:**
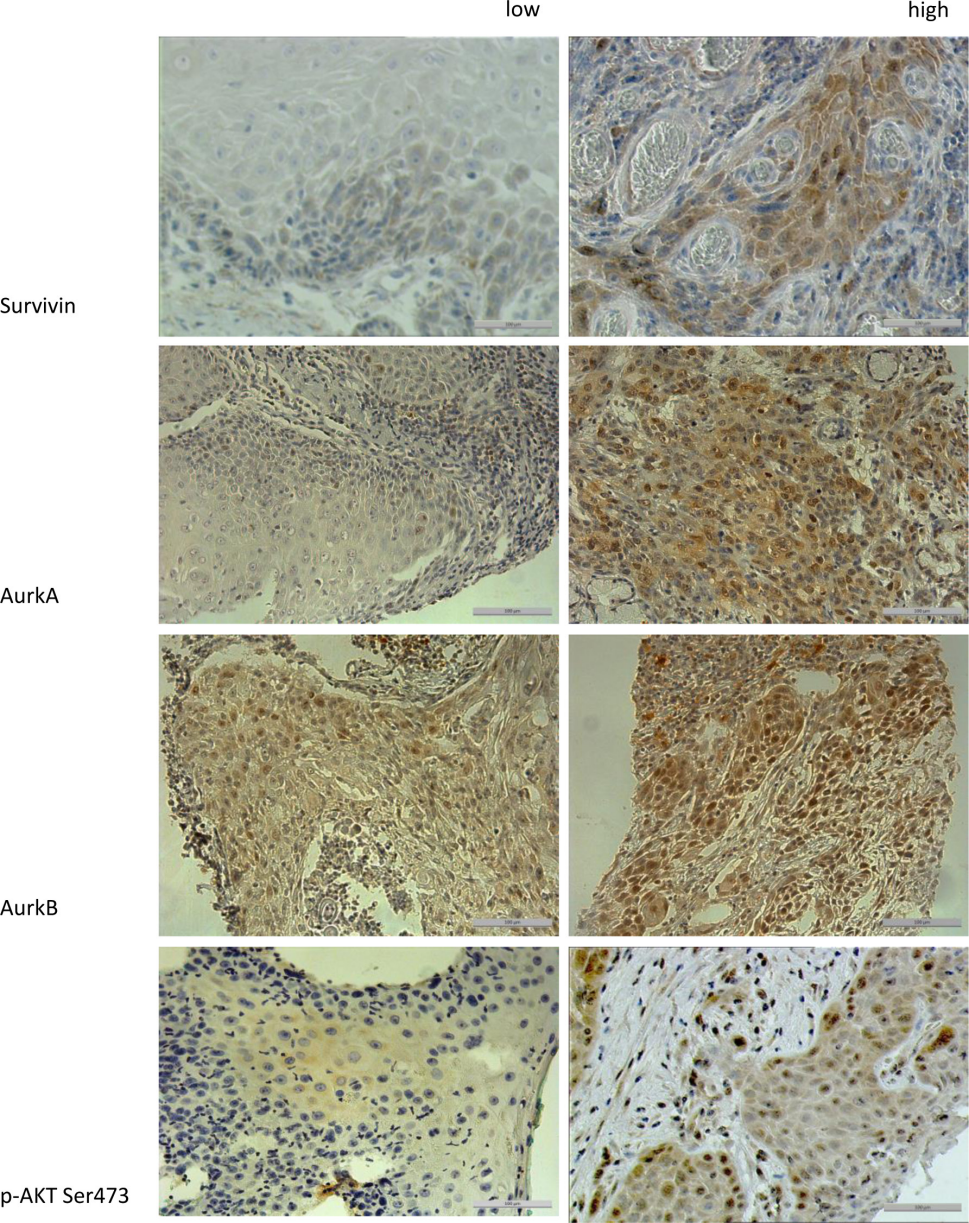
The figure shows the different expression levels (low vs. high) of the proteins Survivin, AurkA, AurkB, and p-Akt Ser 473 in immunohistochemical staining (200× magnification) To compare high with low expression levels, a median split analysis was applied. Marker expression ≥2 were specified as high expression.

In the immunohistochemical staining, the proteins Survivin, p-Akt Ser, AurkA and AurkB showed no correlation with the disease-free survival (all: *p* > 0.1). In the overall survival, only the protein p-Akt Ser 473 expression was correlated significantly (*p* = 0.021) (Figure 1).

Since a number of evidences exists, that indicates an interaction between AurkB and Survivin [9] or AurkA and Protein Kinase B (Akt1) [3], we verified a correlation of combined marker expression by immunohistochemistry (*n* = 182).

High-risk groups with inferior survival rates were found. The high expression of AurkA and Survivin (*p* = 0.020) as well as the combination of AurkA with Akt phosphorylated on Ser 473 (*p* = 0.031) showed a significantly inferior overall survival (Table 1, Supplementary Figure 1).

**Table 1:** Using Kaplan-Meier analysis and the log rank test the prognostic value of individual markers was evaluated

Marker	DFS	OS
AurkA	0.701	0.483
AurkB	0.580	0.571
Survivin	0.311	0.079
p-Akt Ser 473	0.178	***0.021***
AurkA - Survivin	0.107	***0.020***
AurkA - p-Akt Ser 473	0.221	***0.031***
AurkB - Survivin	0.815	0.536
AurkB - p-Akt Ser 473	0.104	***0.041***

High-risk groups have been identified for marker combinations. (DFS = disease free survival, OS = overall survival, italic = statistically significant).

### Homozygous cells show no treatment response to Aurk inhibition unless previously sensitized with docetaxel

**Figure 2:**
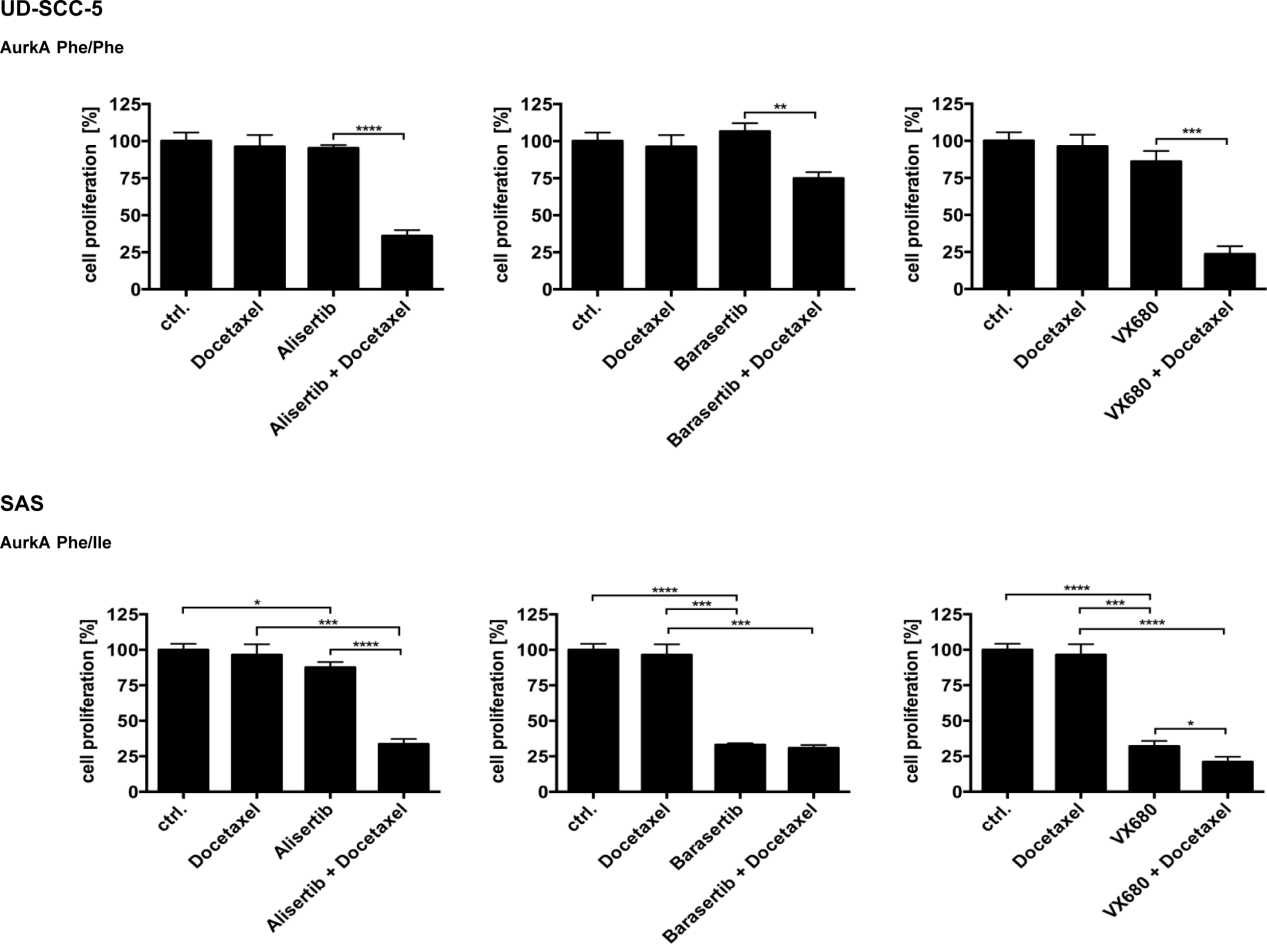
In the proliferation assays, the homozygous cell line showed no significant response to the treatment with the Aurora-Kinase inhibitors Only the adjunction with Docetaxel led to a sensitization and resulted in a significant inhibition of proliferation. In contrast, the heterozygous cell line SAS on the other hand showed a significant response already in the singular treatments with the Aurora-Kinase inhibitors.

The proliferation of the homozygous cell line UD-SCC-5 could not be inhibited by treatment with either Docetaxel, Alisertib, Barasertib, or VX-680. Only a combined treatment of Docetaxel with one of the Aurk inhibitors resulted in a significant inhibition of cell proliferation (Alisertib/Alisertib-Docetaxel *p* < 0.0001, Barasertib/Barasertib-Docetaxel *p* < 0.0014, VX680/VX680-Docetaxel *p* < 0.0003) (Figure 2).

In contrast, the heterozygous cell line SAS showed a significant response to the unimodal treatments with the Aurora-Kinase inhibitors. The effect was most pronounced upon treatment with Barasertib (*p* < 0.0001), followed by VX-680 (*p* < 0.0001). The impact of Alisertib alone was noticeable weaker but still significant (*p* < 0.0192) compared to the other Aurora-Kinase inhibitors. This inhibitory effect was only highly increasable with the addition of Docetaxel for Alisertib (*p* < 0.0001). The proliferation inhibiting effect of VX-680 on SAS was only slightly enhanced in the presence of Docetaxel (*p* < 0.0218), whereas the cells could not be sensitized to Barasertib treatment by exposing the cells to Docetaxel (Figure 2).

### Radiation sensitizes the homozygous UD-SCC-5 cells to Aurk inhibition

**Figure 3:**
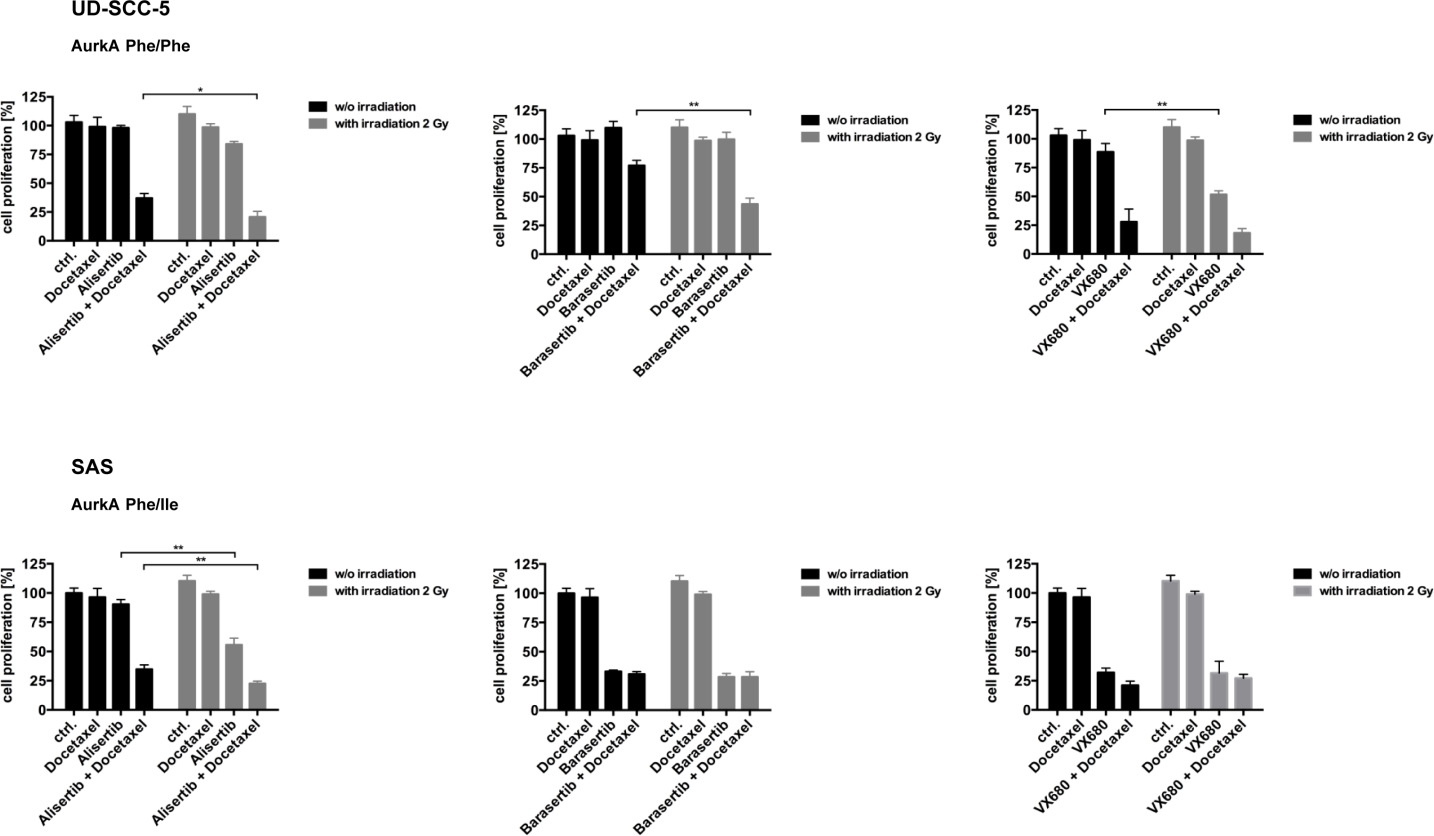
For the homozygous cell line UD-SCC-5 the response to the Aurora-Kinase inhibitors in combination with Docetaxel could significantly be further improved with radiotherapy For the cell line SAS (AurkA Phe/Ile) the supplementary value of radiation was only significant for AurkA inhibition.

The inhibition of UD-SCC-5 cell (AurkA Phe/Phe) proliferation by the combined application of Alisertib and Docetaxel could be significantly enhanced by radiation (*p* < 0.035). The same was true for the combination of Barasertib and Docetaxel with radiation (*p* < 0.0010). A sensitization with radiation alone was found if the cells were treated with VX-680 (*p* < 0.0013) (Figure 3).

Proliferation inhibition of SAS cells (AurkA Phe/Ile) treated with Alisertib alone was significantly enhanced by radiation (*p* < 0.0011). This was liekwise seen when the cells were exposed to both Alisertib and Docetaxel (*p* < 0.0081). Treatment with either Barasertib or VX-680 alone also significantly inhibited cell proliferation. For these two agents, the addition of Docetaxel as well as radiation showed no further effect (Figure 3).

### Aurk inhibition of the heterozygous SAS cells causes polyploidization

**Figure 4:**
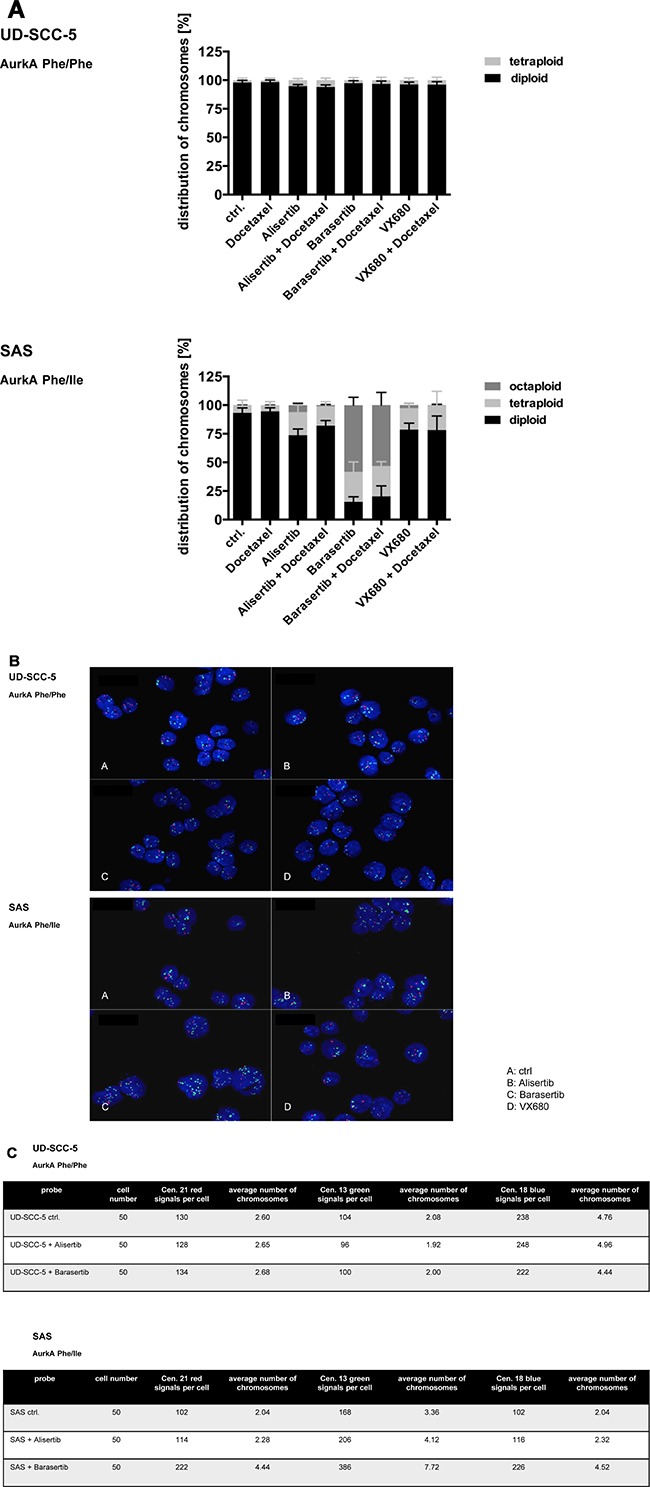
(**A**) Aneuploidy was found with fluorescence-activated cell sorting (FACS) by the heterozygous cell line SAS exhibited a strong increase of tetra- and octaploid cells. The homozygous cell line UD-SCC-5 showed mainly diploid, rarely tetraploid cells. (**B**, **C**) By FISH-analysis an increase of the analyzed chromosomes 13, 18 and 21 for the cell line SAS was seen, especially after the treatment with Barasertib.

Flow cytometric DNA analysis revealed mainly diploid UD-SCC-5 cells. The DNA content was not affected upon treatment with Aurk inhibitors. In contrast, the heterozygous cell line SAS exhibited a strong increase of a tetraploid cell fraction upon treatment with Alisertib and Alisertib/Docetaxel (Figure 4A). The treatment with Barasertib and Barasertib/Docetaxel even resulted in a pronounced fraction of octaploid cells, respectively. Accordingly, fluorescence in-situ hybridization (FISH) revealed a profound degree of polysomy 13, 18, and 21, predominantly upon treatment with Barasertib. (Figure 4B, 4C).

### Exclusively homozygous cells show strong Survivin protein expression

**Figure 5:**
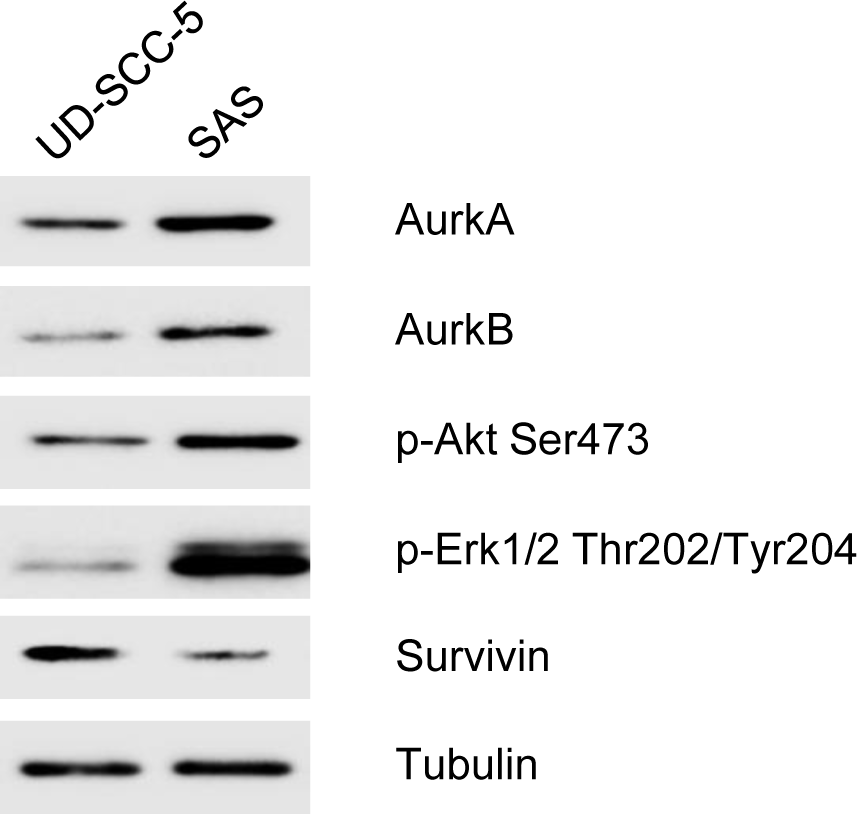
In the Western blot analysis, the homozygous cell line UD-SCC-5 shows a weak expression of Aurora-Kinase A Aurora-Kinase B, p-Akt Ser 473, p-Erk 1/2 Thr 202/Tyr 204 and also expressed strong Survivin In contrast, the cell line SAS shows an increased expression of the Aurora-Kinases A and B as well as p-Akt Ser 473, p-Erk 1/2 Thr 202/Tyr 204. Survivin only showed a very weak expression.

The heterogenous cell line SAS (AurkA Phe/Ile) and the homogenous cell line UD-SCC-5 (AurkA Phe/Phe) differentially expressed AurkA, AurkB, p-Akt Ser 473, pErk1/2 Thr 202/Tyr 204, and Survivin as visualized by Western Blotting. While SAS cells had a strong expression of AurkA, AurkB, p-Akt Ser 473, and pErk1/2 Thr 202/Tyr 204, UD-SCC-5 expressed Survivin at higher levels (Figure 5). Thus, these two cell lines not only inhere different Aurk polymorphisms but also differentially express associated signal transduction proteins.

## DISCUSSION

Conventional therapy regimens of HNSCC include surgery, radiation, and cytotoxic treatments and have not changed considerably during the last years [9]. These treatment modalities, however, do not substantially improve patient's survival or quality of life [10]. The TNM-classification plus the histological tumor grade still represent the only reliable prognostic parameters of the HNSCC disease [11]. Except for the EGFR, prognostic and predictive biomarkers have not been identified so far [11]. Here we analyzed the impact of the AurkA polymorphism on HNSCC cell irradiation response and the effect of specific kinase targeting.

### The prognostic impact of AurkA polymorphism in HNSCC remains controversial

PCR analysis of HNSCC and benign tissues revealed a pronounced expression of the heterozygous AurkA type (Phe/Ile) in tumor samples than in the control cohort [6]. Concordantly, an increased frequency of the heterocygous AurkA genotype in esophageal carcinomas has been found which has been attributed to enhanced tumor progression [7]. The AurkA polymorphism has also been reported for other malignancies as well e.g., breast cancer [12] and hepatocellular carcinomas [13]. Evidently, the AurkA polymorphism variant Phe/Ile has a prognostic (i.e., outcome of disease) and predictive (i.e., impact on treatment with Aurora-Kinase inhibitors) value [14]. However, the prognostic value of the AurkA polymorphism remains controversial. By this study we could not disclose any association of the AurkA polymorphism with HNSCC patient's survival. It has to be considered that radiation was combined with a standard platinum-based chemotherapy in patients and not with docetaxel like in the *in-vitro* assays. Nevertheless, there still appears to be a certain connection, since we found a correlation with the tumor stage.

### Impact of the AurkA polymorphism on the Aurora-Kinase inhibitor mediated sensitization to radiation

Irradiation is a well-established therapy regimen for HNSCC. However, resistance of HNSCC to ionizing irradiation, which probably accounts for relapse or progression, is a clinical problem [15]. Taking this background into consideration we evaluated a possible sensitization to radiotherapy by pre-treatment with different Aurora-Kinases inhibitors.

The response of the HNSCC cell lines to the specific inhibitors of the Aurora-Kinases was validated by concentration kinetics. Kollareddy *et al*. described the characteristics and the limiting factors of the Aurora-Kinase inhibitors used in clinical studies like interactions of the inhibitors with structurally related oncogenic kinases [16]. However, these substances were mainly used for leukemic cell lines (ALL, PALL-2, MOLM13, MV4-11) and solid colon carcinomas, glioblastomas or neuroblastomas. Moreover, an additive effect *in-vitro* for the combination with Docetaxel, a toxin that targets the mitotic spindle, has been reported on an adenocarcinoma cell line of the upper gastrointestinal tract [18] and on esophageal carcinoma cells [17]. For this reason, combination treatment with a simultaneous radiotherapy was also carried out in this work. We observed a sensitization to radiation by Docetaxel as a function of AurkA polymorphism. Interactions between structurally related oncogenic kinases and the Aurora-Kinase inhibitors are considered for altering the action of the inhibitors [16] and a link between the AurkA polymorphism and the applied treatment of the cells (inhibitor and/or sensitizing agent and / or irradiation) could be established.

### Correlation between the Aurora-kinases and aneuploidy

Both Aurora-Kinases A and B are essential for cell mitosis. While the AurkA is important for the centrosome separation, AurkB contributes to the chromosomal passenger complex and thereby controls the chromosomal orientation [8].

It was shown already in 1998 that an AurkA overexpression in mammalian tumor cells can cause aneuploidy [19]. More recent studies indicate an association of elevated AurkA expression levels and a dysfunction of the spindle apparatus which in turn causes a defective cytokinesis [20]. Our data suggest, that predominantly the heterozygous AurkA polymorphism is associated with the polyploidization. Accordingly, heterozygous HN cells (Phe/Ile) but not homozygous Cal27 (Phe/Phe) cells become aneuploid upon irradiation as reported previously [6]. Moreover, the fraction of aneuploid cells can be significantly increased by an AurkB siRNA-knock down. The underlying molecular mechanism, however, remains undiscovered for now.

### Correlation of the Aurora-kinases with Survivin and p-Akt Ser 473

AurkA overexpression has been reported for HNSCC [3] but also for other malignancies [19, 21, 22]. p-Akt Ser 473 as well as Survivin are frequently overexpressed in tumor tissues as well [23]. Based on the survival curve of our patient collective (Supplementary Figure 1) a high-risk group with overexpression of both, AurkA/Survivin and AurkA/pAkt A was identified.

To further clarify the interplay of Aurora-Kinases and the molecules Survivin and p-Akt Ser 473 and to match the *in vitro* and *in vivo* data Western blot analysis was performed. AurkA, AurkB as well as p-Akt Ser 473 and p-Erk1/2 Thr 202/Tyr 204 are overexpressed in the heterozygous cell line SAS. In contrast, the homozygous cell line UD-SCC-5 shows only Survivin upregulation.

Overexpression of Survivin and p-Akt Ser 473 has been previously associated with a poor response to chemo-and radiotherapy and thus with a shortened overall survival [24]. Here we imunohistochemically identified a high-risk collective with elevated AurkA and p-Akt Ser 473. However, it should be noted that low AurkA expression and high p-Akt expression showed the worst survival rates. It was shown that the Aurora kinase A inhibitor Alisertib induces cell cycle G2/M arrest and apoptosis via Akt/mTOR signaling pathways in human breast cancer cells. Despite a low AurkA expression, the blockade with the inhibitors can affect the Akt signal pathway. This inhibition leads to an increased expression of the expression level of membrane-bound microtubule-associated protein 1 light chain 3 (LC3)-II and beclin 1 and induced inhibition of protein kinase B (Akt). This could be a possible explanation for why the inhibition of the AurkA is also justified in this case [25, 26].

Additionally, a high AurkA and p-Akt Ser 473 expression was exclusively found in the heterozygous cell line SAS. Hence, it is plausible, that in this cell line the treatment with Docetaxel or irradiation did not enhance the inhibitory effect of Barasertib or VX-680.

## CONCLUSIONS

Our results suggest that the AurkA polymorphism is probably not a prognostic factor for HNSCC patient's survival. Further studies should retrospectively and prospectively address the therapy response as a function of AurkA polymorphism in the clinical setting. This could define patient stratification more precisely and consequently enhance the treatment outcome.

## MATERIALS AND METHODS

### PCR-restriction fragment length polymorphism (RFLP)

DNA was isolated from cell lines and the FFPE tumor (*n* = 115) samples using the DNeasy Kit (Qiagen, Hilden, Germany) according to the manufacturer's instructions.

The specific PCR for AurkA genotypes at the Phe_31_Ile site was performed using the Taq-DNA Polymerase All-inclusive Kit (PeqLab, Erlangen, Germany) according the manufacturer's guidelines. We applied the following thermocycler (BioRad, Munich, Germany) PCR programme: (95°C for 5 min, 30 cycles of 95°C for 30 s, 60°C annealing for 30 s, and 72°C for 30 s), and 72°C for 7 min. The following primers were used:

AurkA forward, CTTTCATGAATGCCAGAAAGT T, and reverse, CTGGGAAGAA-TTTGAAGGACA.

The 165-bp PCR products were digested with ApoI (New England BioLabs, Inc., Beverly, USA) and separated on a 2.5% agarose gel. Digestion of the AurkA 31Phe allele results in two fragments (153 bp and 12 bp), whereas the AurkA 31Ile allele contains an additional third ApoI restriction site and thus three bands (89 bp, 64 bp, and 12 bp) are generated after digestion. The results were further confirmed by DNA sequencing using an ABI 3100 DNA sequencer (Life Technologies, Darmstadt, Germany).

### Tissue samples

Formalin-fixed and paraffin-embedded tumor samples of 182 patients (mean age 54.27 years, range 30–70 years) with a squamous cell carcinoma of the oral cavity (*n* = 33), oropharynx (*n* = 59), hypopharynx (*n* = 34) or larynx (*n* = 56) were examined regarding Aurora-Kinase A and B, Survivin, and p-Akt Ser 473 expression. The samples were collected during the period 1993–2006 in the Klinikum rechts der Isar, Technical University Munich and University of Regensburg (Table 2).

**Table 2:** Clinical characteristics of patients included in the study

Characteristica	Data	
**Age**		
average age	54,27 years	
range	30–70 years	
**Sex**		
male/female	165/17	90.7%/9.3%
**TNM-classification**		
**pT-calssification**		
pT1	26	14.3%
pT2	66	36.3%
pT3	49	26.9%
pT4	41	22.5%
**pN-classification**		
pN0	91	50.0 %
pN1	25	13.7%
pN2a	2	1.1%
pN2b	45	24.7%
pN2c	19	10.4%
pN3	0	0%
**cM-classification**		
cM0	182	100%
M1	0	0%
**Grading**		
G1/G2/G3	10/110/62	5.5%/60.4%/34.1%
**Localisation**		
oral cavity	33	18.1%
oropharynx	59	32.4%
hypopharynx	34	18.7%
larynx	56	30.8%

The Medical Ethics Committee of the Technical University of Munich approved this study (project number 1420/05).

### Immunohistochemical study

Fresh 1.5 μm sections were transferred to glass slides, deparaffined and rehydrated. Antigen retrieval method (microwave oven heating in citrate buffered saline) was applied following the instructions provided by the manufacturer. After cooling down the TMA slides were incubated with the following antibodies: Aurora-Kinase A (rabbit, clone 1F8) 1:200 (Cell Signaling Technology, Frankfurt, Germany), Aurora-Kinase B (rabbit) 1:200 (Cell Signaling Technology), Survivin (rabbit, clone 12C4) 1:100 (Dako Agilent Technologies, Hamburg, Germany), p-Akt Ser 473 (rabbit, clone 736E11) 1:20 (Cell Signaling Technology). The reaction was developed with the labeled streptavidin-biotin-peroxidase system. DAB was used as the reaction indicator. After counterstaining with hematoxylin, slides were dehydrated in ascending concentrations with ethanol and mounted. As positive control tissues with known expression of the respective antigen were used. For negative control, irrelevant antibodies with the immunoglobulin isotypes were used.

### Scoring system for protein expression in immunohistochemically staining

A scoring system was used to describe the expression levels of the different proteins. Staining intensity was graded from 0 to 3 points (0 points = no staining, 1 point = low staining intensity, 2 points = moderate staining intensity, 3 points = strong staining intensity). The proportion of stained cells was estimated and graded from 0 to 4 points (0 points = 0% of the tumor cells, 1 points = <10% of the tumor cells, 2 points = 10%–29% of the tumor cells, 3 points = 30%–59% of the tumor cells, 4 points = 60%–100% of the tumor cells). Both scores were added to form a cumulative score. All scoring analysis was done by two independent investigators. To compare high with low expression levels, a median split analysis was applied. Marker expression ≥2 were specified as high expression.

### Cell culture

The cell line SAS was obtained from DSMZ (Braunschweig, Germany). The cell line UD-SCC-5 was provided from the University of Düsseldorf (Clinic for Otolaryngology, Düsseldorf, Germany).

Cells were cultured in Dulbecco's modified Eagle medium (DMEM) (Invitrogen, Darmstadt, Germany) containing 10 % fetal calf serum (FBS) (Biochrom, Berlin, Germany), 2 mM glutamine, 100 μg/ml streptomycin and 100 U/ml penicillin (Biochrom) and maintained at 37°C in an atmosphere of 5 % CO_2_ grown to a 70–90 % confluence.

### Inhibitors

All inhibitors Alisertib, Barasertib, VX-680 and Docetaxel were obtained from Selleckchem (Darmstadt, Germany). After concentration kinetics, we chose Alisertib in a concentration of 5 nM to inhibit Aurora-Kinase A activity. Barasertib was used in a concentration of 1 nM to block Aurora-Kinase B. Cells were treated with 2.5 nM VX-680 to inhibit Aurora-Kinase A and B. The blocking agent of mitosis, Docetaxel was medicated in a concentration of 0.25 nM (Supplementary Figure 2).

### Radiation

Irradiation of cells was performed using an X-ray generator (Gulmay CP-2225; maximum dose rate 3 Gy/min) at the Department of Radiotherapy (Technische Universität München). Cells were irradiated 24 h after seeding at room temperature (70 kV, 10 mA) with X-ray doses between 0.5 and 16 Gy at 70 kV and 10 mA using a filter #3 and a table of 466 mm height. This resulted in a dose of approximately 1 Gy/min. Control cells were treated accordingly, however without irradiation. Supplementary Figure 3 shows the kinetics rays for the two cell lines.

### Crystal-violet proliferation assay

Cell survival was assessed by crystal-violet assay. Cells were seeded in 6-well tissue culture dishes (5 × 10^3^ cells/well). After prior treatment with Aurora-Kinase inhibitors for 48 h, cells were irradiated with 2 Gy. After 10 d cells were subsequently formalin fixed and visualized by crystal violet staining (Sigma-Aldrich, Steinheim, Germany). For staining the cells, culture medium was aspirated and 500 μl of 4% formaldehyde was added to each well for 30 min. After washing with 0.1% Triton-X100/PBS and H_2_O, crystal violet (0.04%) was added to the fixed cells and allowed to act for 30 minutes. Finally, SDS (1%) was added and the optical density was measured at 590 nm using an ELISA reader 1 h later.

### Western blot

For protein expression analysis cells were lysed in 1× lysis buffer (New England Biolabs, Frankfurt, Germany) supplemented with 1 mM PMSF (Roth, Karlsruhe, Germany). Equal amounts of protein (15 μg) were separated by SDS-PAGE and transferred to Immobilion membranes (Millipore, Schwalbach, Germany). Blocking of unspecific binding sites was performed using 5% (w/v) non-fat dry milk in TBST. Membranes were incubated with primary antibodies diluted in TBST for 12–14 hours at 4°C. HRP-conjugated immunoglobulins (diluted 1:5000 in 5% non-fat dry milk/TBST) served as detection antibodies and were probed for 1 h at room temperature. Immunoreactivity was visualised by exposure to high-performance chemiluminescence film (G&E Healthcare, Freiburg, Germany). We used primary antibodies against p-Akt Ser 473 (rabbit, clone D9E) 1:500 (Cell Signaling), p-Erk1/2 Thr 202/Tyr 204 (rabbit) 1:1000 (Cell Signaling), Survivin (rabbit) 1:1000 (Cell Signaling), Aurora-Kinase A (rabbit) 1:500, Aurora-Kinase B (rabbit) 1:500 and Tubulin (rabbit) 1:5000.

### Aneuploidy analysis by flow cytometry

24 h after seeding in culture flasks (75 cm^2^, 5 × 10^5^ cells per flask) UD-SCC-5 and SAS cells were subjected to a combined treatment regimen as described above. 48 h after treatment cells were detached by trypsinization, washed twice with ice-cold PBS containing 2% FCS and fixed/permeabilized with 70% methanol on ice for 60 min. Finally, cells were washed twice with PBS and resuspended in PBS containing 1 μg/ml DAPI 15 min prior to analysis. 1 × 10^5^ DAPI stained cells of every sample were analyzed using a BD FACSCanto-II^TM^ Flow Cytometer (BD Biosciences, San Jose, USA). DNA histograms were plotted on a linear scale and cell cycle fractions, i.e. percentages of cells in G0/G1-, S- and G2/M-phase, were quantified using the ModFit LT 3.2 software (Verity Software House, Topsham, ME, USA) upon cell doublet, aggregate, and debris discrimination via pulse processing.

### Fish

Fluorescence *in situ* hybridization was performed using the hybridization buffer ZytoLight SPEC 13/CEN 18/SPEC 21 Triple Color after the application the manufacturer's guidelines of ZytoVision GmbH (Bremerhaven, Germany) and analyzed by the epifluorescence microscope Axio Imager Z1 (Zeiss, Oberkochen, Germany). Before the actual hybridization 5 × 10^4^ cells of the already fixed and treated cells are done on a microscope slide. Then 50 cells were counted and their fluorescent signals were analyzed.

### Statistical analysis

Statistical analyses were performed using the GraphPad Prism software, version 6.0. Assuming a symmetry correlation structure for all experiments, all hypotheses were tested with the One-way ANOVA test. Using a *t*-test, means of treated cells and untreated controls were compared. The level of statistical significance was set at *p* < 0.05.

To evaluate the clinical data, the SPSS software 23 was used. Using Kaplan-Meier analysis and the log rank test the prognostic value of individual markers was evaluated. Correlation analysis for the clinical data (T-classification, lymph node metastasis, distant metastasis) was performed using a cross analysis/four-field table (chi-square test, Fisher's exact test).

## SUPPLEMENTARY MATERIALS FIGURES


