# Supplementary material for: The Aurora-Kinase A Phe31-Ile polymorphism as possible predictor of response to treatment in head and neck squamous cell carcinoma
Source: Oncotarget. 2018 Jan 30;9(16):12769–80. doi: 10.18632/oncotarget.24355 (PMC5849172; doi:10.18632/oncotarget.24355)
Supplement: Supplementary file 1 [file oncotarget-09-12769-s001.pdf]

The Aurora-Kinase A Phe31-Ile polymorphism as possible predictor of response to treatment in head and neck squamous cell carcinoma

SUPPLEMENTARY MATERIALS

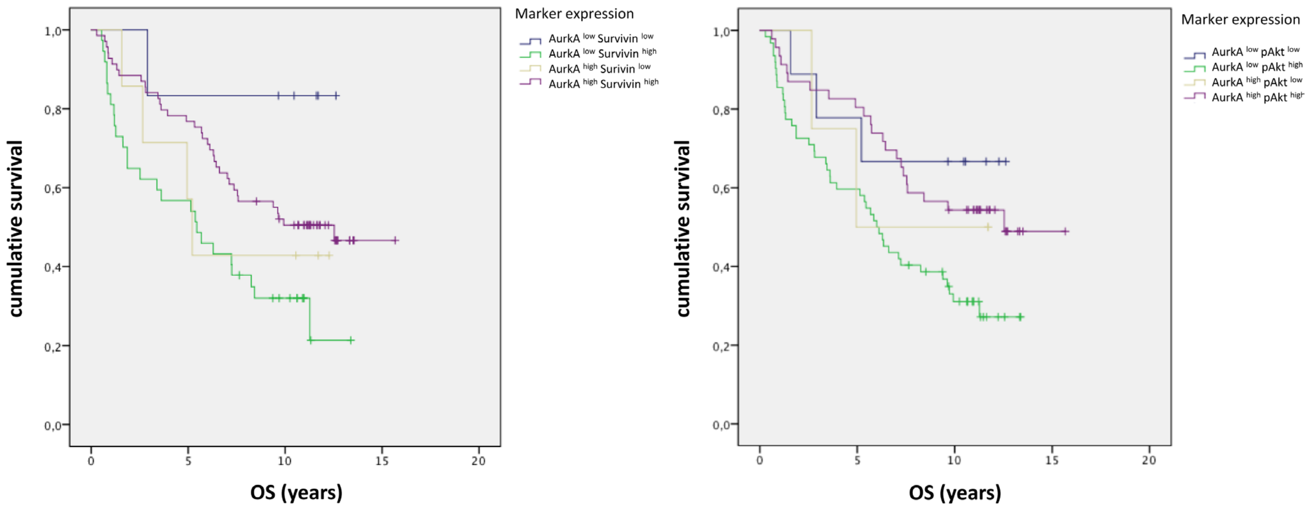

Supplmentary Figure 1: The difference in overall survival of patients with AurkA^{high} and Survivin^{high} is statistically different from the survival of patients who are characterized by AurkA^{low} and Survivin^{low}. $p = 0.02$. The same applies to AurkA and pAkt, $p = 0.031$. The staining score is defined in the material and method section.

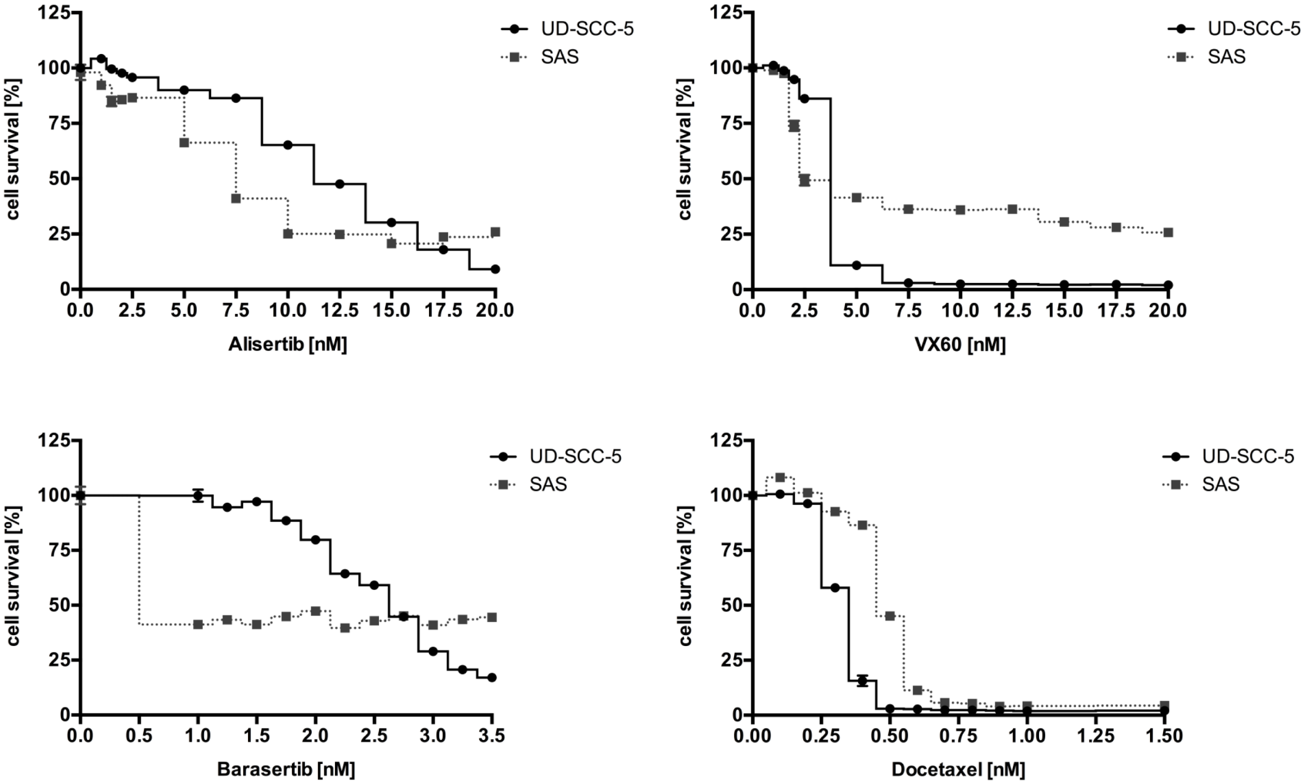

Supplmentary Figure 2: Concentration kinetics of Alisertib, an AurkA inhibitor, Barasertib, an AurkB inhibitor, VX-680, inhibit Aurora-Kinase A and B, and Docetaxel were shown.

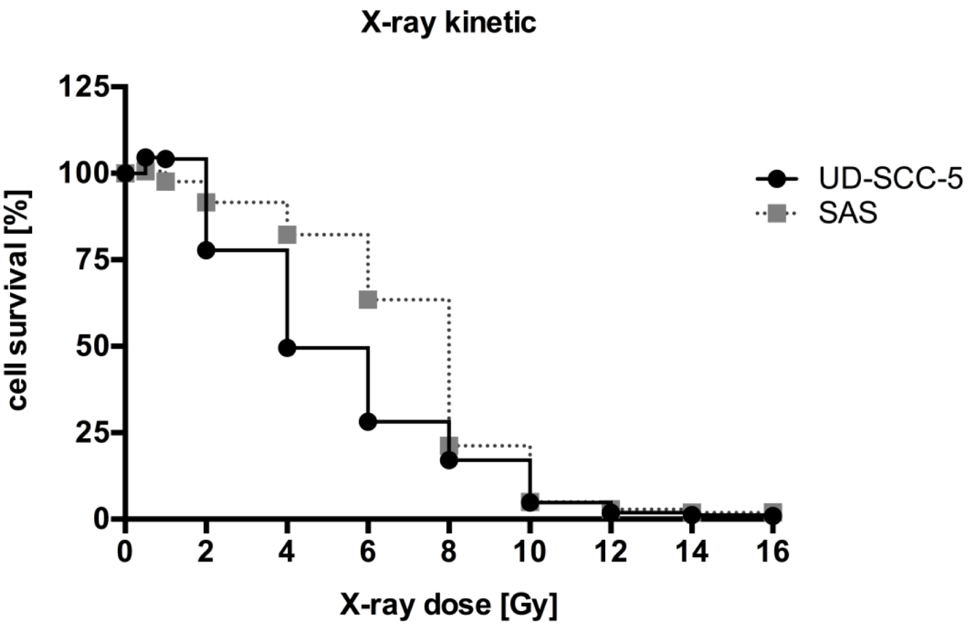

Supplmentary Figure 3: The figure shows the kinetics rays fort the two cell lines.
